# Chloroplast genome structure and phylogenetic position of *Calanthe sylvatica* (Thou.) Lindl. (Orchidaceae)

**DOI:** 10.1080/23802359.2019.1642157

**Published:** 2019-07-18

**Authors:** Li-Yuan Miao, Chao Hu, Wei-Chang Huang, Kai Jiang

**Affiliations:** a Shanghai Chenshan Plant Science Research Center, Chinese Academy of Sciences, Chenshan Botanical Garden, Shanghai, China;; b College of Life and Environmental Sciences, Shanghai Normal University, Shanghai, China

**Keywords:** *Calanthe sylvatica*, Illumina sequencing, chloroplast genome, phylogenetic

## Abstract

*Calanthe sylvatica* plays an important role in horticulture and is undergoing the pressure of population decline. In this study, its complete chloroplast genome sequence was successfully obtained by the technology of Illumina sequencing. The whole chloroplast genome length was 158,828 bp with a typical quadripartite structure: one large single copy (LSC) region (87,277 bp), one small single copy (SSC) region (18,535 bp) and a pair of inverted repeats (IR) regions (26,508 bp). The GC content of this genome was 36.7%. The genome contained 118 genes consisting of 84 protein-coding genes, 30 tRNA genes, and 4 rRNA genes. The phylogenetic analysis indicated a close relationship between *C. sylvatica* and *Calanthe triplicata*.

The orchid *Calanthe sylvatica* is found in scattered areas of Asia and Africa. It is of high horticultural value due to the various forms and unique patterns of its blooms (Su et al. 2012). Wild populations of this species are decreasing sharply as the result of over-acquisition and habitat destruction. The first volume of the China Species Red List listed all species of *Calanthe* as either critically endangered or endangered (Wang and Xie 2004). Increased knowledge concerning this plant’s genomic makeup may provide insights into its effective conservation (Hu et al. 2018). Unfortunately, genetic information for *C. sylvatica* is still quite limited. In this study, we sequenced the complete chloroplast genome of *C. sylvatica* using next-generation technology.

Fresh leaves were collected from adult *C. sylvatica* plants distributed in Malipo, Yunnan Province, China (23.19°N, 104.47°E) and the specimen stored at the Shanghai Chenshan Botanical Garden Herbarium (CS-HWC201607-10). Genomic DNA was extracted using the Plant Genomic DNA Kit (TIANGEN: DP305-03) and the purified DNA was then used to construct libraries, which were sequenced using the Illumina MiSeq platform. A total of 1.78 G of clean data were obtained by trimming off low-quality bases and adaptor-ligated regions using Trimmomatic v0.32 (Bolger et al. 2014). The complete chloroplast (cp) genome annotation of *C. sylvatica* was performed using Dual Organellar GenoMe Annotator (DOGMA) software (Wyman et al. 2004). This annotation was manually corrected for the start/stop codons and intro/exon boundaries by comparison to homologous reference genes in the homologous species of *Calanthe triplicata* (NC_024544). We then submitted the complete annotated cp genome sequence to GenBank and received accession number MK736029.

The complete cp genome sequence of *C. sylvatica* was found to be 158,828 bp in length, with a characteristic circular structure consisting of a pair of inverted repeats (IRs) (26,508 bp) separated by SSC and LSC regions (87,277 bp and 18,535 bp, respectively). The guanine-cytosine (GC)-content of this genome is 36.7%. There are a total of 118 genes in the genome, namely, 84 protein-coding genes, 30 tRNA genes, and 4 rRNA genes, containing 20 duplicated genes. Of the 20 duplicated genes in the IR regions, 8 are protein-coding genes, 8 are tRNA genes, and 4 are rRNA genes. Fifteen genes have introns, namely, 9 protein-coding genes and 6 tRNA genes. There are 3 genes with 2 introns each, namely, *clpP*, *ycf3*, and *rps12*. Specifically, the *rps12* is a trans-spliced gene with its 5′ end exon located in the LSC region, while the intron 3′ end exon is situated in the IR region.

To clarify the phylogenetic position of *C. sylvatica*, we then constructed a maximum-likelihood tree using the complete cp genome sequences of 28 orchid species with MEGA6 software (Tamura et al. 2013). According to the phylogenetic tree (Figure 1), we determined unambiguously that there is a closer relationship between *C. sylvatica* and *C. triplicata* than with *Calanthe davidii*.

**Figure 1.**
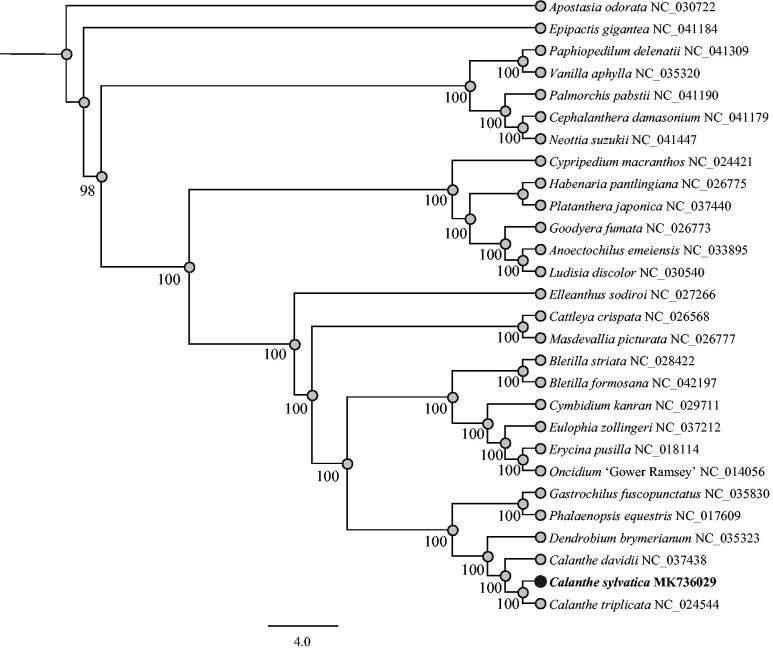
Phylogenetic relationship of 28 orchidaceae species based on the full cp genome sequences with maximum-likelihood (ML) analysis.

## Geolocation information

Malipo, Yunnan Province, China (23.19°N, 104.47°E)
